# Alternating Currents

**Published:** 1902-08

**Authors:** 


### ALTERNATING CURRENTS.

### UNCLE HENRY ON PASSING OF THE HORSE.

Every little while they tell us that the horse has got to go ;
First the trolley was invented, 'cause the horses went so slow,
And they told us that we'd better not keep raisin' colts no more ;

When the street cars got to moting that the horses pulled before
I thought it was all over for old Fan and Doll and Kit,
S'posed the horse was up and done for,
But
he
ain't
went
yit.

When the bike craze first got started people told us right away,
As you probably remember, that the horse had saw his day.
People put away their buggies and went kitin' round on wheels ;
There were lots and lots of horses didn't even earn their meals.
I used to stand and watch 'em, with their bloomers as they'd flit,
And I thought the horse was goin',
But
he
ain't
went
yit.

Then they got the horseless carriage, and they said the horse was done,
And the story's been repeated twenty times by Edison ;
Every time he gits another of his batteries to go,
He comes whoopin' out to tell us that the horse don't stand a show,
And you'd think, to see these chauffeurs, as they go a-chauffin', it
Was good-by to Mr. Dobbin,
But
he
ain't
went
yit.

When the people git to flyin' in the air, I s'pose they'll say,
As we long have been a-sayin', that the horse has had his day,
And I s'pose that some old feller jist about like me'll stand
Where it's safe and watch the horses haulin' stuff across the land,
And he'll mebby think as I do, while the crowds above him flit,
"Oh, they say the horse is done for,
But
he
ain't
went
yit."

